# Deep social neuroscience: the promise and peril of using artificial neural networks to study the social brain

**DOI:** 10.1093/scan/nsae014

**Published:** 2024-02-08

**Authors:** Beau Sievers, Mark A Thornton

**Affiliations:** Department of Psychology, Stanford University, 420 Jane Stanford Way, Stanford, CA 94305, USA; Department of Psychology, Harvard University, 33 Kirkland St., Cambridge, MA 02138, USA; Department of Psychological and Brain Sciences, Dartmouth College, 6207 Moore Hall, Hanover, NH 03755, USA

**Keywords:** artificial neural network, deep learning, primer, computational, methods

## Abstract

This review offers an accessible primer to social neuroscientists interested in neural networks. It begins by providing an overview of key concepts in deep learning. It then discusses three ways neural networks can be useful to social neuroscientists: (i) building statistical models to predict behavior from brain activity; (ii) quantifying naturalistic stimuli and social interactions; and (iii) generating cognitive models of social brain function. These applications have the potential to enhance the clinical value of neuroimaging and improve the generalizability of social neuroscience research. We also discuss the significant practical challenges, theoretical limitations and ethical issues faced by deep learning. If the field can successfully navigate these hazards, we believe that artificial neural networks may prove indispensable for the next stage of the field’s development: deep social neuroscience.

Social neuroscience resides at the confluence of two torrents of complexity: the raging river of neural complexity and the deluge of social complexity. Either of these currents could sweep a researcher off their feet. To survive this flood, we often grasp at the riverbanks—either simplifying the neuroscientific questions we ask, to better grapple with the complexity of the social world, or simplifying the social phenomenon we consider, to better understand the complexity of the brain. Neither solution is entirely satisfactory. Simplified neuroscientific approaches may not yield a useful understanding of the brain (Jolly and Chang, 2019; Jonas *et al*., 2017). Simplified social stimuli and tasks may not scale up or generalize to the rich social questions we intrinsically care about (Zaki and Ochsner, 2009; Schilbach *et al*., 2013; Wheatley *et al*., 2019). But what if we could swim against the current, taking on the complexity of both the social brain and social environment simultaneously? We propose that artificial neural networks (ANNs) can support research that embraces both neural and social complexity.

This review offers a primer on ANNs tailored to social neuroscientists. Our goal is to help you build useful intuitions for how ANNs work and to orient you to promising applications and important limitations. In so doing, we aim to deflate some of the unjustified hype surrounding deep learning, while also giving you an appreciation of its genuine merits. First, we introduce basic concepts and terminology in a survey of the building blocks of ANN architectures. Second, we detail three major use cases for neural networks: improving statistical models, annotating social stimuli and behavior and creating cognitive models of social processing. Third, we outline the practical, theoretical and ethical limitations of ANNs. We conclude by discussing the role that neural networks may play in shaping the future of social neuroscience.

## Primer

### Components of ANNs

**Fig. 1.**
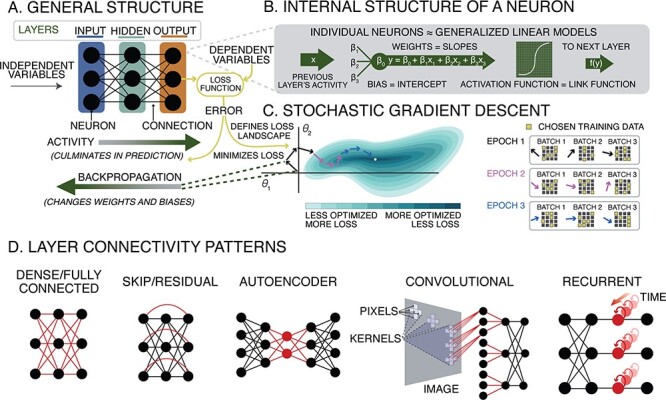
(A) The general structure of an ANN, with elements shared by all architectures indicated. (B) The internal structure of each individual neuron resembles a generalized linear model. (C) The optimization process of stochastic gradient descent, along with training terminology. (D) Common layer connectivity patterns—red elements indicate the defining features of each.

This primer aims to give social neuroscientists who are already familiar with linear and logistic regression a set of building blocks they can use to conceptualize their own neural network architectures. To achieve that goal, we first must review the basic components of neural networks and how they are trained to accomplish specific tasks (Figure 1).

ANNs consist of nodes that represent neurons, and edges that connect nodes together, representing synapses (Figure 1A; Table 1). Similar to how biological neurons transmit electro-chemical signals, nodes and edges in a neural network transmit numerical signals: nodes are activated when they receive signals from input edges, all inputs are summed, a bias parameter is added and the result is transformed using a non-linear activation function (similar to a link function in a generalized linear model). The result is then sent to downstream nodes via output edges, each of which multiplies the signal by a weight parameter. This process may be more familiar to psychologists than it first appears: given a sigmoid (logistic) activation function, a single node performs logistic regression. By chaining together many logistic regression-like transformations, ANNs can implement arbitrarily complicated functions (Hornik *et al*., 1989).

**Table 1.** Translating the terminology of deep learning

Terms used by social neuroscientists	Deep learning equivalents
Vector	First order tensor
Matrix	Second order tensor
(Multidimensional) array	Higher order tensor
Independent variable(s)	Feature(s)
Dependent variable(s)	Target(s) (‘labels’ for categorical variables)
Total error/log-likelihood	Loss/cost
Link function	Activation function
Regression coefficients (slopes)	Weights
Regression coefficients (intercept)	Biases
(Generalized) linear model	Neuron
Model	Network
Fitting	Learning
Measurement error	Noise
Dummy coding	One-hot encoding
Measurement invariance	Concept drift
Principal components analysis	Autoencoder (linear activation function)
Manifold learning	Autoencoder (non-linear activation)
Standardization/z-scoring	(Batch) normalization
Generalization	Transfer learning
Prediction	Activation (of output layer)
Chance	Baseline

Most ANNs in current use are deep networks, which are organized in layers. Signals flow from an input layer with one node per data feature or independent variable, through any number of intermediate hidden layers, each with many nodes, terminating at a final output layer with one node per output feature or dependent variable. Prototypically, nodes in each layer receive signals only from nodes in the previous layer and send signals only to nodes in the next layer. When every node in a layer sends output to every node in the next layer, the network is said to be fully connected or ‘dense’. Although fully connected networks are widely used, many interesting network architectures use their own idiosyncratic layer arrangements. This modular attitude is a key feature of the research culture of ANNs: progress in neural network methods often consists in modifying or swapping out some part of a network, observing that it works better than expected, and then trying to figure out why. This inverts conventional statistical practice, which often proceeds from mathematical proof to practical application.

### Training and optimization

Training a neural network is an optimization problem: we want to find the edge weight and node bias parameters that produce the best possible performance on a given task. This search requires both a measure of current performance and a method for updating the parameters. Conceptually, this is similar to fitting regression weights. But because a neural network consists of many non-linear operations chained together, a more involved training procedure is required (Figure 1C).

Task performance is measured using a loss (or objective) function that compares the network’s predictions to ‘correct’ or desired answers (labels). The closer the network’s predictions are to the labels, the lower (better) the value of the loss function. For example, regression tasks often use mean squared error loss, which is the average of the squared differences between the network’s predictions and the ground truth. Binary classification tasks often use cross-entropy loss (log loss), which is the logarithm of the difference between the labels (1 or 0) and the network’s prediction. Although these two loss functions may be familiar from linear and logistic regression, neural networks can make use of any loss function, and novel loss functions are often designed for specific tasks.

Neural networks are typically initialized with random weight and bias parameters. Training the network consists in changing those parameters to minimize the chosen loss function. In practice, neural networks are usually trained using gradient-based optimization algorithms such as stochastic gradient descent. Gradient descent is conceptually straightforward: iteratively adjust the parameters to follow the gradient (slope) of the loss function to a local minimum. Analogously, the quickest way to the bottom of a hill is to follow the steepest path.

The gradient of the loss function is typically estimated using the backpropagation algorithm (Rumelhart *et al*., 1995). Backpropagation begins with a forward pass through the network. An item from the training set activates nodes in the network’s input layer, which each sends activations to nodes in the next layer, and so on until the output layer. A backward pass—moving from the output layer to the input layer—calculates how much the activations in each layer need to change to get the correct output. This process yields the gradient of the loss function with respect to the network parameters, given a single training example. Backpropagation is an application of the chain rule from calculus and can be expressed in terms of matrix operations that are efficient to compute on consumer hardware.

After backpropagation, the parameters are moved in the direction of the gradient, scaled by a chosen step size, decreasing the expected value of the loss function. Over many repetitions, the network’s randomly initialized parameters are massaged into place, minimizing the loss function and producing output approximating the training data. When the gradient is calculated using all available training data at once, this is called batch gradient descent. In contrast, when the gradient is iteratively approximated by randomly drawing single items from the training set, this is called stochastic gradient descent. Mini-batch gradient descent lies between these two extremes, averaging the estimated gradients across limited-size samples or ‘mini-batches’ of the training data. The latter offers a balance between efficiency and accuracy that makes it the most popular method.

The process described above applies to most neural network architectures. However, the details of how a model is trained can have a large effect on the speed of training and the quality of the results. The training needs to take into account both network architecture and target task, and finding the right approach can be difficult (Gotmare *et al*., 2018).

### Overfitting

Like many machine learning algorithms, neural networks are prone to overfitting: performing well on their training data but failing to generalize to new data. This is in part because neural networks typically have many parameters—sometimes more than there are training examples—allowing them to ‘solve’ problems by simply ‘memorizing’ all the input–output pairings presented during training (Zhang *et al*., 2019). Several techniques are used to minimize the risk of overfitting.

One familiar technique is cross-validation (Browne, 2000). The training data are split into subsets, one of which is held out as a validation set, used only to evaluate the performance of the network, but never used to update the network’s parameters. For cross-validation to be effective, it is crucial to prevent data leakage: that is, there must be no information about the specific validation observations in the training set. If, during training, the loss decreases on the training set, but increases on the validation set, this is a sign of overfitting. Cross-validation supports early stopping of the training process when overfitting is observed (Caruana *et al*., 2000).

Neural network inputs are typically normalized (standardized) to have zero mean and a standard deviation of 1. This ensures that the dimensions of the input space all have the same scale, improving the loss landscape (Figure 1C) by smoothing out steep ravines into wider, rounder basins, making it easier for the gradient descent algorithm to escape local minima. The same logic applies within the network. Normalizing each layer’s output has a regularizing effect, ensuring that no parameters grow too large, and further smoothing the loss landscape. Within the network, the mean and variance of each sample must be calculated on a per-batch basis, and so this process is referred to as batch normalization (Ioffe and Szegedy, 2015; Santurkar *et al*., 2018).

Although it is possible to regularize ANN weights by adding a penalty term to the loss function (like the L2 norm in ridge regression), it is more common to use dropout, where randomly selected sets of neurons are removed (dropped out) from the network at each stage of training (Srivastava *et al*., 2014). As with regularization by limiting large weights, dropout limits the network’s ability to rely on a single path through the network for producing any given result.

### Good performance despite overfitting

Neural networks often generalize better than one might expect, given the large number of parameters. Most statistical models are subject to a bias–variance tradeoff: models that are too simple (that are highly biased) will underfit the data, whereas models that are too complex will overfit the data, resulting in generalization error (high variance) (Geman *et al*., 1992). The bias–variance tradeoff account predicts a U-shaped generalization error curve: models that have too many or too few parameters should have high error, while models with just the right number of parameters should have low error.

Rather than showing a U-shaped error curve, neural networks often show a ‘double descent’ curve. As parameters are added, error decreases to a local minimum, then increases following a U-shape, before suddenly and consistently decreasing (Belkin *et al*., 2019). The transition between increasing and decreasing error has been described as a phase transition separating two qualitatively different regimes: an underparameterized regime where neural networks are subject to a bias–variance tradeoff and where overfitting must be managed using early stopping, dropout, etc., and an overparameterized regime where the bias–variance tradeoff does not apply and performance simply improves as additional parameters are added. Successful overparameterized neural networks behave like minimum-norm interpolating classifiers, estimating where test inputs lie in a smooth latent space capturing the training data (Liang and Recht, 2021). On this account, neural networks are manifold learners that guess what should be ‘in between’ training data points. However, it has also been argued that the second descent in error is simply evidence of a second U-shaped curve caused by a bias–variance tradeoff involving a set of neurons in the network that learn at a slower rate (Pezeshki *et al*., 2022).

### Building blocks of deep neural networks

#### Input and output, softmax and multilayer perceptrons

This section aims to build intuition about what ANNs can do and how they do it, giving the reader a set of modular building blocks they can use to design neural networks suited to their research goals (Table 2). We start with input and output layers, describing a very simple neural network equivalent to regression and classification models. We then proceed to modules that can support complex tasks like image generation, natural language processing and playing complex games.

**Table 2.** Conceptual building blocks for neural networks

Name	Description	Application
Input layer	Layer with one node per IV	Provide input
Output layer	Layer with one node per DV	Read network output
Activation function	Function applied at each node before the signal is sent to downstream nodes	Like a link function in a generalized linear model; usually non-linear
Loss function	Measures error on a given task	Used in optimization to find parameters that support task performance
Fully connected layer	Layer connected to all nodes in previous and next layers	Transform inputs to support creating the desired outputs
Softmax layer	Activations across all nodes normalized to sum to 1	Tasks where the desired output is a probability distribution
Hidden layer	Layer between the input and output layers	An intermediate processing stage or representation; deeper networks have performance advantages
Multilayer perceptron (MLP)	Network with only fully connected layers, usually with one or more hidden layers	Universal function approximation (including classification and regression)
Embedding layer	Fully connected layer, usually with fewer nodes/lower dimension than its input	Find a low-dimensional approximation of input (an embedding)
Residual block	Series of layers running in parallel with other layers	Process data in more than one way at the same time
Skip/shortcut connection	Edge that bypasses one or more layers	Use a representation in earlier layers in a later processing stage
Encoder	Series of layers with progressively lower dimension	Compress data in stages, discarding redundant information
Decoder	Series of layers with progressively greater dimension	Reconstruct compressed data in stages, recovering discarded information
Autoencoder	Encoder connected to a decoder	Middle layer learns a low-dimensional manifold representing the training data; typical architecture for generative uses
Reconstruction loss	Loss function measuring the difference of the output and the input	Train network to accurately represent training data; used with autoencoders
Convolutional neural network	Imitates human vision using many convolution and pooling layers	Represent visual features independent of spatial position
Convolution layer	Convolves/cross-correlates input with a learned filter	Used with pooling layers to implement visual receptive fields
Pooling layer	Calculate a summary statistic over input	Used with convolution layers to implement visual receptive fields
Recurrent feedback connection	Edge from a later layer to an earlier layer	Implement ‘top-down’ processing for individual inputs; represent context when processing sequences of inputs
One-hot encoding vector	Binary vector where each register corresponds to a single input or input part and only one register is set to 1	Represent inputs or input parts that are qualitatively different; e.g. words in a vocabulary
Multi-hot encoding vector	Like a one-hot encoding vector, but may have any number of registers set to 1	Represent qualitatively different and simultaneously occurring inputs or input parts; like a bag-of-words
Long short-term memory network (LSTM)	Network module that uses recurrent connections for sequence processing	Tasks where the output depends on a sequence of preceding inputs; e.g. next word prediction
Sequence-to-sequence (seq2seq)	Sequence encoder paired with a sequence decoder; an autoencoder for sequences	Tasks where both inputs and outputs are sequences; e.g. natural language translation, question answering, text generation
Transformer	Network architecture that uses attention and positional encoding for sequence processing	Tasks where the output depends on a sequence of preceding inputs; e.g. next word prediction; handles long-distance dependencies better than LSTMs
Attention	Representing each target token as a weighted transformation of all source tokens	Used in transformers to handle long-distance dependencies
Positional encoding vector	A vector added to transformer inputs that ‘tags’ tokens with information about their absolute and relative positions	Used in transformers to handle long-distance dependencies
Hybrid architecture	A model using ANNs alongside non-neural components	Used to overcome the limitations of ANNs
Pre-trained network	A network trained and released for public use	Often more practical than designing and training an ANN from scratch
Fine-tuning	Further training a pre-trained network on a new task	Adapting pre-trained networks for specific use-cases, e.g. dataset annotation

An ANN composed of only two layers can perform linear regression: an ‘input layer’ with one node per independent variable (IV) (e.g. survey answers), fully connected to an ‘output layer’ with one node representing the dependent variable (DV). In practice, most ANNs use non-linear ‘activation functions’, but to implement linear regression, all nodes should use a linear activation function. To minimize the squared error (as in ordinary least squares regression), the network is trained with a least-squares ‘loss function’ and all nodes use a single shared bias parameter. The network’s edge weights correspond to regression coefficients and the bias parameter corresponds to the regression intercept (Figure 1B).

The structure of the training data must match the input and output layers: training datasets typically consist of many pairs of tensors (a 0-dimensional tensor is a scalar, 1d is a vector, 2d is a matrix, etc.), with input tensors representing the values of each IV and output tensors representing the corresponding values of each DV. Each value in the input tensor is fed to a corresponding node in the input layer, and each node in the output layer is compared to the corresponding value of the output tensor when calculating the loss.

With minor modifications, the ANN described above can perform many related regression and classification tasks. For example, using a single-node output layer with a logistic activation function will cause the network to perform logistic regression, similar to using a logistic link function in a generalized linear model. Importantly, changes to the format of the output layer often require corresponding changes to the loss function. Least-squares loss measures the overall error across all DVs, but the binary, true-or-false error of logistic regression is better measured using a cross-entropy loss. Multi-class classification can be achieved by adding a node to the output layer for each target class. To obtain a probability distribution over possible outputs, the output layer can be changed to a ‘softmax layer’ that scales the activation values of all nodes such that they sum to 1, producing a probability distribution. The node with the maximum softmax value corresponds to the predicted class of the input.

One natural extension of this architecture is the ‘multilayer perceptron (MLP)’: use a non-linear activation function, and add a fully connected layer between the input and the output layers, often called a ‘hidden layer’. MLPs are expressive tools for regression and classification because they are universal function approximators (Hornik *et al*., 1989)—they can approximate any distribution of training data, given a sufficient number of independent samples. This property of fully connected layers is often used to find a low-dimensional approximation of network’s input—or an ‘embedding’*—*that is used by later layers. When it serves this purpose, a fully connected layer is sometimes called an ‘embedding layer’.

Adding additional nodes (greater width) or additional layers (greater depth) will increase the capacity of an MLP, allowing it to learn a more accurate approximation—but note that wide and deep networks have different performance characteristics (Nguyen *et al*., 2020). Deep networks are often more practically effective than wide networks, but deep networks are susceptible to vanishing or exploding gradients: as backpropagation chains together many matrix multiplications, the values passed through the network tend to exponentially increase to the maximum or decrease to zero. Exploding gradients are often addressed using gradient clipping, which simply limits gradient values. Vanishing gradients are often addressed using ‘residual blocks’ containing several conventional neural network layers that run in parallel with a ‘skip or shortcut connection’ that bypasses one or more intermediate layers. These skip connections prevent gradients from vanishing and allow the network to maintain and use a representation of its input (or the output of earlier layers) at later processing stages.

#### Autoencoders and generative networks

Social psychologists often perform factor analysis on survey measures: survey questions that tend to be answered the same way are understood as representatives of a shared latent factor, and the survey can be compressed by choosing a subset of questions per factor. ‘Autoencoders’ are ANNs that are particularly good at finding compressed, latent representations of their inputs (Bank *et al*., 2020). This goes not only for survey measures, but for any domain where a complicated surface hides a simpler underlying structure. For example, we experience human faces not as complicated lists of pixel color values, but as arrangements of constituent parts (eyes, mouths, noses and so on). Autoencoders excel not only at finding latent factors, but also at using those factors to generate realistic items that mimic their training data. For example, when trained on a large database of face images, an autoencoder can create a low-dimensional ‘face space’ which can be used to generate realistic faces not present in the training data.

Autoencoders have two parts: an ‘encoder’ consisting of many layers, each with fewer nodes than the previous, followed by a ‘decoder’, also consisting of many layers, each with more nodes than the previous (Figure 1D). The central layer with the fewest nodes (the last layer of the encoder or the first layer of the decoder) constitutes an information bottleneck—the training data must be compressed to fit, or embedded, within that layer (Tishby and Zaslavsky, 2015). Typically, autoencoders are trained using a ‘reconstruction loss’ function that measures how well the output matches the input, such that an output identical to its corresponding input has zero loss. Reconstruction loss encourages the encoder to discard redundant information before the bottleneck or embedding layer, and encourages the decoder to reconstruct the input based on its compressed representation, recovering the discarded redundant information. This process is similar to factor analysis, with two key differences: (i) non-linear activation functions allow the network to capture non-linear latent factors, typically resulting in a more accurate representation; (ii) instead of learning factors that share variance, the network learns factors that discard redundancies without compromising reconstruction.

Autoencoders can be used to generate novel items by activating the bottleneck layer directly, picking an arbitrary point on the embedding manifold and sending it to the decoder, causing it to ‘reconstruct’ an item not present in the training data. It can be useful to imagine the autoencoder creating a new item by optimally mixing the relevant features of nearby training items, taking non-linear interdependencies between features into account. Interestingly, training an autoencoder to ‘fill in the blanks’ in natural language text can yield a word embedding that captures many interesting semantic properties; e.g. analogies can be represented as spatial transformations in the embedding space (Mikolov *et al*., 2013).

Autoencoders share an important limitation with factor analysis: nodes in the bottleneck layer do not necessarily correspond to human concepts. As a consequence, the latent factors found by autoencoders can be difficult to interpret. Variational autoencoders address this limitation by using a probabilistic representation (Doersch, 2016), and are often regularized so the network learns independent probability distributions, disentangling the underlying latent factors to make them more amenable to human interpretation (Burgess *et al*., 2018).

As noise is essentially information irrelevant to reconstruction, autoencoders are remarkably good at denoising data. This is accomplished by training the autoencoder using noisy data, but calculating the loss based on noiseless data—effectively training the autoencoder to reconstruct images without any noise (Vincent *et al*., 2008). Denoising autoencoders can be used to (among other things) learn orientation-independent representations of faces and objects, to mitigate measurement error in noisy domains such as fMRI and to realistically upscale images to higher resolutions.

#### Convolutional networks for image processing

Analysis of visual images poses many tricky problems. One is feature integration: how do we know that a specific pattern of light hitting our retina means we see a cat? Another is position invariance: how do we learn that a cat is a cat regardless of whether it’s on the left or right of an image? Learning to identify a ‘cat’ from the pixel values of an image is a lot like identifying a latent factor in a survey—or rather, it would be, if cats always appeared in exactly the same position in every image. Learning position invariance—recognizing a cat no matter where it appears—requires extra work.

One naive solution could be to use an extremely large training set, in which cats of every shape and color appear in every possible part of the visual field. But this leads to a combinatorial explosion, as the amount of required training data scales with the number of features multiplied by the number of possible spatial positions. Further, the number of network parameters scales multiplicatively with the image size. ‘Convolutional neural networks’ seek to avoid these combinatorial explosions by adding layers designed to represent visual features independently from their spatial positions (Fukushima, 1980; LeCun and Bengio, 1995).

Convolutional networks were inspired by the receptive fields of human visual perception. Visual features are detected using filters that operate over each receptive field, and which are implemented as a ‘convolution layer’. Each node in a convolution layer has input edges from a subset of input nodes that define its receptive field. The weights of these edges are shared across receptive fields, defining a convolutional filter used as a feature detector (Figure 1D). The output of a convolution layer is the input convolved or cross-correlated with the learned filter, allowing convolutional networks to learn visual features that best support performance on the training data. Convolutional networks often include ‘pooling layers’ that reduce dimensionality by calculating a summary statistic (such as the maximum or the average) for each receptive field. By stacking convolution and pooling layers, convolutional networks implement a perceptual hierarchy, where simple feature detectors that capture edges and corners are combined to capture complex features.

Further architectural innovations include the use of residual blocks and ‘recurrent feedback connections’ that allow the network to use representations from one processing stage at earlier or later processing stages. These innovations make convolutional networks much better at image classification tasks than fully connected networks, suggesting that hierarchical feature detection across receptive fields is an efficient architecture for visual learning. Accordingly, convolutional neural networks are often good models of visual cortex (Liao and Poggio, 2016).

#### LSTMs and transformers for sequence processing

Natural language is highly context-dependent. For example, ‘Let’s eat, grandma!’ is innocuous in comparison to the sinister ‘Let’s eat grandma!’ How do neural networks represent the difference?

Conventionally, words are represented using a ‘one-hot encoding vector’: a vector of all zeros equal to the size of the vocabulary, but containing a single 1 at a position corresponding to the represented word. A straightforward way to combine the words of a sentence is to sum the one-hot vectors of each word, creating a ‘multi-hot encoding vector’ or bag of words. Although the bag of words approach is simple, it does not capture word order—the multi-hot vector for ‘dog bites man’ will be the same as that for ‘man bites dog’. The need to capture ordering has led to major innovations in neural network architecture, including recurrence and attention.

‘Long short-term memory networks’ (LSTMs) are designed to process sequences of inputs, where previous items in the sequence are stored in memory and used as context for evaluating the current item (Hochreiter and Schmidhuber, 1997). This is accomplished using recurrent connections, where the output of each sequence step is fed back into the network and represented in parallel with the next input sequence step, continuously maintaining memory of the sequence context (Figure 1D). By adjusting layer weights during training, LSTMs can learn strategies for transforming input into memory and input + memory into output. The maintenance of context memory supports tracking dependencies between inputs at different sequence steps. In many cases, LSTMs are arranged in a ‘sequence-to-sequence’ (seq2seq) architecture similar to an autoencoder: a set of stacked LSTM layers encodes the input sequence and outputs a context memory vector, and another set of stacked LSTM layers decodes that context vector into an output sequence. LSTM seq2seq models perform remarkably well on tasks like natural language understanding, translation and speech recognition. However, recurrent maintenance of context memory has an important limitation: the further apart two tokens are, the more likely an intervening step will disrupt the context memory that tracks their dependence.

‘Transformers’ address this limitation by tracking long-distance dependencies with ‘attention’ and ‘positional encoding’ (Vaswani *et al*., 2017). Because sequence-to-sequence LSTMs build a context memory by moving step-by-step over the input, longer sequences are subject to accumulating noise and vanishing gradients. In contrast, transformers use an attention mechanism to represent each token in the input as a complicated weighted transformation of all the other tokens at once, capturing many non-linear dependencies between tokens. Learning attention weights that minimize the loss is like learning how to look at different parts of a sentence as you read through it; for example, inferring who is referred to as ‘them’ by looking earlier in the sentence for their name. By adding a positional encoding vector to every input, each token is ‘tagged’ with a value representing both its absolute and relative positions. Because attention and positional encoding allow transformers to represent long-distance dependencies more crisply, they can exceed the performance of LSTMs on sequence-based tasks like natural language processing. When trained on very large datasets, the performance of transformers can be remarkable: the success of large language models like GPT4 (Achiam *et al*., 2023), as well as text-to-image models like Stable Diffusion (Rombach *et al*., 2022), can be partially attributed to the transformer architecture.

#### Hybrid architectures

Theories about how people interact are increasingly explored using agent-based simulations, where many software agents interact and their emergent behavior is measured. ANNs can play an important role in such simulations, often as a normative model of optimal learning. To use ANNs in this way, they must be integrated into a larger system with non-neural parts: a ‘hybrid architecture’. Hybrid architectures take advantage of the unique strengths and weaknesses of their components, compensating for the limitations of isolated neural networks. Importantly, hybrid architectures sometimes succeed on reasoning and planning tasks where LSTMs and transformers fall short.

One fruitful innovation has been the incorporation of deep neural networks into reinforcement learning systems (Sutton and Barto, 2018). This suggests that the state spaces of many RL tasks support interpolation, and are thus better represented as a smooth latent space in the embedding layer of a neural network. Deep RL agents are particularly good at playing games, with a single model approaching or exceeding human performance at 49 different Atari games (Mnih *et al*., 2013). Interestingly, many hybrid architectures make extensive use of randomness. AlphaGo, the first machine learning model to achieve superhuman performance at the game of Go, depended on Monte Carlo Tree Search (Silver *et al*., 2016) to explore possibilities that might otherwise be overlooked.

Many important problem domains require manipulating symbols according to clearly defined rules. For example, determining the truth value of a proposition in symbolic logic requires tracking how the truth values of its constituent parts are manipulated by logical operators. In such symbolic problem domains, small changes in the input can produce large changes in the output, and approximations are often inappropriate: answers are either exactly right or unusably wrong. For these reasons, symbolic systems are not a natural fit for conventional ANNs. However, hybrid architectures can combine ANNs with program synthesis tools, improving performance on symbolic tasks (Balog *et al*., 2016). Typically, an ANN is used to embed an input, and that embedding conditions a search through a well-defined space of possible programs that could yield the correct output. This hybrid approach has better performance on problems involving symbolic logic, and has also been used to model human concept learning (Feinman and Lake, 2018; Ellis *et al*., 2021).

#### Pretrained networks and fine-tuning

As evidenced by the breadth of material covered in this primer, constructing a neural network architecture from scratch is time- and resource-intensive, creating a high barrier to entry. But there is a cheaper, easier way: download a pretrained network from a public repository (e.g. HuggingFace: https://huggingface.co/), and then optionally fine-tune the network on a desired task. This approach is often used to test the flexibility of models trained on very large datasets (Devlin *et al*., 2018; Radford *et al*., 2019). For example, a researcher who wants to use an ANN to annotate natural language responses from study participants could download a transformer neural network model that had been pre-trained to perform next-word prediction on an extremely large database of publicly available text. Then, they could fine-tune the model by performing additional training on the desired annotation task. This works because many of the representations learned by models trained on very large databases tend to be task-general: convolutional neural networks learn basic visual features like corners and edges that can be repurposed to recognize objects only presented during fine-tuning training. Likewise, transformer large language models learn basic semantic features that can be repurposed for annotation, sentiment analysis and so on.

Although fine-tuning a pre-trained model is practical in many situations, this approach has several important limitations. Critically, the provenance of the training data may be difficult to verify, making it difficult to assess bias and raising concerns about whether data were collected in accordance with ethical standards (Birhane and Prabhu, 2021). Further, using pre-trained models can make it difficult or impossible to perform experiments where the architecture or training datasets vary across conditions, limiting the potential of pre-trained models for researchers using ANNs as models of brain activity or cognitive processes.

## Applications of ANNs in Social Neuroscience

**Fig. 2.**
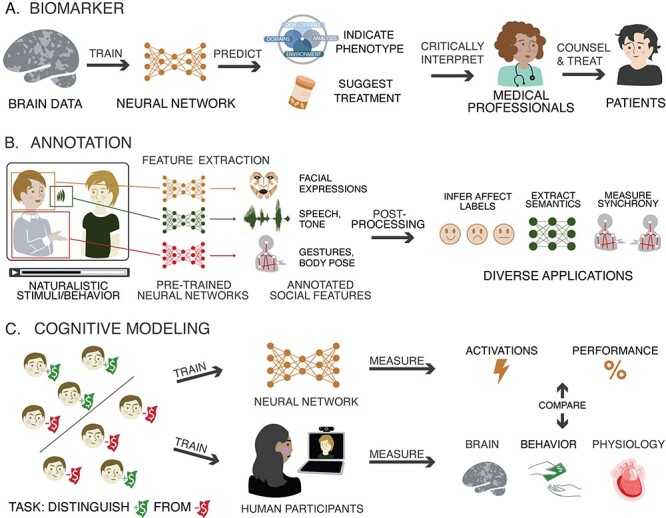
Major applications of artificial neural networks to social neuroscience: (A) Deep neural networks can serve as statistical models to predict phenotypes or suggest treatments based on brain data; (B) Pre-trained ANNs can automatically annotate a wide range of socially relevant features from audiovisual data; (C) ANNs can serve as cognitive models, learning the same task as human participants and then modeling their behavior, physiology or neural representations.

We next review three broad categories of applications for ANNs in social neuroscience (Figure 2). First, they can create better predictive models linking brain structure or activity to behavior or other real-world outcomes. Second, they can annotate, or generate, naturalistic stimuli or participant behavior. Third, ANNs can model cognitive or neural processes. In this section, we will explore these three categories of applications, give existing examples of each, and outline their future promise.

### Building better predictive models

A major focus of human neuroscience in recent decades has been the search for biomarkers of mental illness and neurological conditions. The ultimate goal of this research is to develop predictive models that can take in data such as MRI scans, and make clinically useful predictions about diagnoses, trajectories or treatment efficacies (García-Gutiérrez *et al*., 2020).

Clinically useful biomarkers for complex mental health conditions such as mood and anxiety disorders have thus far proven elusive (Strawbridge *et al*., 2017). This is particularly true for fMRI-based biomarkers, which have been a major focus of social neuroscience research. With a few exceptions, such as the neurologic pain signature (Wager *et al*., 2013), fMRI has not yielded biomarkers with sufficient reliability, sensitivity and specificity to prove clinically useful. Might ANNs help us make progress in this direction?

Until recently, most biomarker studies relied on linear models. These models cannot capture non-linear associations between brain and behavior, such as a situation in which either too much or too little activity in a brain region predicts clinical dysfunction. Fortunately, deep neural networks are well-suited to learning many different types of non-linear relationships (Figure 2A) while avoiding the risks of overfitting (Liang and Recht, 2021). Convolutional neural networks in particular are well-suited to classifying image data (He *et al*., 2015). Applying such models to neuroimaging data eliminates the burden of manual feature engineering while still offering the capacity to capture rich non-linear relationships between brain and behavior.

Researchers are already starting to apply ANNs to develop diagnostic models based on fMRI data (Yin *et al*., 2022). In recent years, attempts have been made to develop deep learning based predictive models of cognitive impairment (Wen *et al*., 2018), attention deficit hyperactivity disorder (Mao *et al*., 2019; Riaz *et al*., 2020), Alzheimer’s disease (Sarraf and Tofighi, 2016) and autism spectrum disorder (Li *et al*., 2018), among other conditions. Although not specifically assessing ANNs, recent research does suggest that multivariate models are generally more reliable predictors of behavior from brain data (Marek *et al*., 2022). As a result of this promise, we anticipate that more biomarker research will gravitate towards the use of ANNs. However, ANNs suffer from certain limitations which may forestall this approach—in particular, they are data hungry, difficult to interpret and often learn misleading ‘shortcuts’ instead of causally necessary relationships—as we describe later in this article.

There is widespread consensus in the neuroscientific community that existing diagnostic categories do not reflect the underlying structures or mechanisms of mental health conditions (Casey *et al*., 2013). Exploratory models like unsupervised/self-supervised ANNs may reveal new ways to conceptualize disorders of the mind and brain. Unsupervised ANNs could also serve more pragmatic purposes. For example, autoencoders could be used to compress neuroimaging data (Makkie *et al*., 2019). Unsupervised generative models of brain data could also be used to produce large synthetic datasets for algorithm development or *in silico* experimentation (Ramezanian-Panahi *et al*., 2022; Jain *et al*., 2023).

### Annotating, manipulating and generating naturalistic stimuli and behavior

Human social life is inherently complex, contextual and dynamic (Vallacher *et al*., 2002; Cikara *et al*., 2022). The average social neuroscience experiment is not. Even mundane conversations teem with possibilities, ambiguities and flux. In contrast, most neuroimaging studies consist of participants alone in the dark pressing buttons to respond to what resembles a slideshow of images or text. There are good reasons why most social neuroscience studies bear little resemblance to daily life. Non-naturalistic designs offer tight experimental control that justifies strong causal inferences. Their temporal structures also typically permit efficient and relatively straightforward modeling of hemodynamic responses. We have learned—and doubtless will continue to learn—a great deal through these experiments.

However, the balance between naturalistic and non-naturalistic research is arguably skewed. We spend little of our time studying phenomena, such as conversations, that make up the bulk of real social interaction. Recent commentaries have called for rebalancing research by shifting focus onto understanding these naturally-occurring social phenomena (Zaki and Ochsner, 2009; Schilbach *et al*., 2013; Wheatley *et al*., 2019). Given these calls, why does naturalistic interaction-focused social neuroscience remain relatively rare? There are likely multiple contributing factors, but here we will focus on one: the difficulty of grappling with the complexity of naturalistic stimuli and behavior (Wheatley *et al*., 2023). How does one take something as complex as a conversation, and convert it into meaningful numbers susceptible to statistical analysis?

Historically, the main answer to this question has been manual coding. People watch recordings of naturalistic stimuli or participant behavior, and annotate what is happening. Although this approach can be highly effective, it is inherently costly in terms of time, effort and often money. This lack of scalability makes it difficult to collect the large samples that may be necessary to quantify social heterogeneity across diverse individuals, contexts and cultures.

ANNs offer a solution to this problem (Figure 2B). Over the past decade, ANNs have made remarkable progress at performing many of the functions once performed by manual annotators. Speech can be automatically transcribed to text (Fortuna and Nunes, 2018), and the text converted to vectors representing semantic features (Devlin *et al*., 2018). The movement of muscles in the face, or the limbs of the body, can be measured from ordinary video recordings (Cheong *et al*., 2021; Kocabas *et al*., 2021). The identities of people and objects can be tracked over time and through space (Kuo and Nevatia, 2011). Pre-trained models capable of performing these annotations are increasingly accurate, open-source and user-friendly, and can be fine-tuned to particular applications. With affordable hardware, they can be run at near real-time speeds, opening new horizons for the interactive use of such data in naturalistic experiments. In this way, the rise of ANNs dovetails perfectly with the increasing interest in interaction neuroscience, making it possible to study naturalistic social interactions in ways, and at scales, that simply would not have been possible before. In addition to annotations, ANNs such as autoencoders and generative adversarial networks can manipulate existing stimuli or generate novel stimuli.

Already ANNs have been applied to annotate and generate stimuli in support of neuroscience research. Language models can quantify semantic features of words in conversations (Goldstein *et al*., 2022), the actions of characters in naturalistic movies (Thornton and Tamir, 2020, 2022) and participants’ facial expressions while they watch them (Chang *et al*., 2021). Generative networks are being trained to produce novel faces for the purposes of manipulating first impressions of traits (Peterson *et al*., 2022). These applications only scratch the surface of what these tools can potentially do for the field, as recent strides in research with non-human animals helps to show (Lauer *et al*., 2022; Pereira *et al*., 2022). However, there are important caveats to these uses of ANNs, including both their generally lower accuracy compared to human annotators, and their potential for reproducing human biases. We detail these concerns later in this article.

### Cognitive and neural modeling

Neuroscientists strive to develop better models of the brain. With the advent of ANNs, vision science and allied subfields have undergone a modeling revolution (Richards *et al*., 2019; Hasson *et al*., 2020). In particular, convolutional neural networks have been applied to model a wide range of visual behavior and the corresponding neural representations (Conwell *et al*., 2022). These models have proven highly effective, achieving some of the highest model fits to fMRI data to date in visual cortex, approaching ceiling performance. That said, the degree and nature of the fit between ANNs and organic brains is still a matter of active research and debate (Lonnqvist *et al*., 2021; Saxe *et al*., 2021; Bowers *et al*., 2022; Guest and Martin, 2023; Momennejad, 2023).

The use of ANNs as cognitive models is only just beginning to spread to social neuroscience (Bolotta and Dumas, 2021). Neural responses to narratives and other linguistic content are being modeled using deep learning language models (Dehghani *et al*., 2017; Hu *et al*., 2022; Zada *et al*., 2023). Visual deep nets are being used as models against which the explanatory power of social features can be compared (Dima *et al*., 2022; McMahon *et al*., 2023). Simple ANNs are being used to understand the information and goals that shape emotion concepts (Thornton *et al*., 2023). ANNs are also being used to model the development of self-concept (Noguchi *et al*., 2022). As the use of ANNs in social neuroscience matures, we expect to see an even broader range of phenomena explored using this method (Figure 2C). To the extent that ANNs faithfully represent the functional principles of brain activity, they may serve as test beds for *in silico* experiments that could not be carried out on biological brains (Wang *et al*., 2022; Jain *et al*., 2023).

Another way ANNs could be useful to social neuroscience is by capitalizing on one of their limitations: the reproduction of human biases. As we discuss below, ANNs reflect and reproduce social biases in their training data. If one’s goal is to quantify and understand bias, then this otherwise problematic property could prove to be an asset. Society-level bias is already being measured using large language models (Charlesworth *et al*., 2021). Others have applied deep neural networks to better understand the biased ways in which humans judge one another’s faces (Peterson *et al*., 2022).

How might the use of ANNs in social neuroscience differ from that in other subfields? Deep reinforcement learning models may be particularly important as cognitive models in social neuroscience because they can be used to model interactions between agents, and the mental representations generated to subserve those interactions. Researchers can develop deep reinforcement models through interactions between humans and ANN agents or between ANNs and other ANNs (Holcomb *et al*., 2018). Although single-player and competitive multiplayer games have thus far been the focus of this research, examination of cooperative interactions has also begun (OroojlooyJadid and Hajinezhad, 2023).

ANNs can also be of particular use to social neuroscientists by allowing us to build cognitive models of multiple interacting brains. Recent years have seen a rapid increase in the use of hyperscanning to study social interactions, and a proliferation of computational methods for analyzing the resulting data (Hakim *et al*., 2023). These methods are now starting to receive convenient software implementations (Ayrolles *et al*., 2021). ANNs such as transformers may provide flexible ways of measuring interbrain coupling. Unlike many extant methods, these ANNs make no assumptions about the linearity or time-locked nature of the relationships between brains, and they can scale up from dyads to larger groups. Moreover, they can operate in the time domain, frequency domain or both simultaneously. This property makes them excellent candidates for harmonizing cognitive models across the neuroimaging modalities which are popular for hyperscanning. These methods, including fMRI, electroencephalography (EEG), magnetoencephalography (MEG) and functional near infrared spectroscopy (fNIRS), differ greatly in their spatial and temporal resolutions. However, ANNs could learn to embed data from these different modalities into a common latent space, thereby allowing researchers to capitalize on the unique advantages of each method while mitigating their respective limitations.

## Limitations of ANNs

Despite their promise, ANNs have many limitations—some unique, and some shared with other machine learning algorithms. In this section we will explore the limitations of ANNs in detail. We will also show how these limitations are not purely technical, as they interact with important procedural, social and ethical considerations.

### Dataset size

ANNs, like other models with large numbers of parameters, need large amounts of training data to learn and generalize effectively. Exactly how much data depends on factors such as the complexity of the problem being solved and the architecture of the network. For example, the ImageNet dataset—which jumpstarted the deep learning revolution in computer vision—consists of over a million labeled images of 20 000 categories of objects (Deng *et al*., 2009). More recent image and text datasets used to train state-of-the-art computer vision and language learning models are far larger (OpenAI, 2023). This practical limitation has theoretical implications: deep neural networks need more data than human learners to achieve similar performance (Liu *et al*., 2021). This may be because ANNs lack the inductive biases that constrain and accelerate human learning (Goyal and Bengio, 2020).

The amount of data required by deep learning creates practical challenges. The cost of obtaining data is likely to be a particularly important consideration for social neuroscientists. Collecting enough fMRI data to train a clinically useful deep learning biomarker is likely beyond the capabilities of any one lab. Consortia and other large team projects are the only feasible way forward for such an approach (Kandel *et al*., 2013). The use of other methods including EEG, MEG and fNIRS may help address this limitation of fMRI.

Large datasets can cause problems even after they are collected. The hardware needed to store the data and train models on it can become expensive, as can the associated electricity, physical space, personnel and other infrastructure costs. The best performing large language and image models are now impractical to train for any but the wealthiest organizations. Although communities can crowdsource distributed training of large models intended for general use (Ryabinin and Gusev, 2020), this can only partially close the gap, as scientists will often desire models trained on specific data and tasks. Fine-tuning or compressing existing large ANNs may help to mitigate this issue (Dodge *et al*., 2020; Zhu *et al*., 2023).

### (In)accuracy

The performance of ANNs varies widely from task to task. ANNs already outperform humans at some tasks, such as protein folding or playing certain games (Mnih *et al*., 2013; Jumper *et al*., 2021). For other tasks—such as objection identification—ANNs perform about as accurately as human coders (He *et al*., 2015). However, for many tasks, ANNs perform worse than humans and may exhibit distinctly non-human behavior. Researchers seeking to use ANNs should consider what level of fault-tolerance their application allows for. How many errors—and what type of errors—are permissible in exchange for the scalability and rapidity of ANNs? In some cases, any error might be intolerable, but in many cases, even moderate accuracy could prove useful. Putting a ‘human in the loop’ to identify and correct errors in ANN output may mitigate inaccuracy (Wu *et al*., 2022). For example, a speech-to-text model can provide an initial transcript of a conversation, and then a human can manually correct its errors, resulting in considerably less work than transcription by hand.

ANNs often fail when the distribution of data used in training is somehow different from the distribution on which it is applied. This is called distribution shift (Quinonero-Candela *et al*., 2008). Generalization failures caused by distribution shifts are perhaps the most common failure mode of ANNs, and a major barrier to practical applications. Distribution shifts are likely to affect social neuroscience researchers in two cases: in translational or predictive applications, including the detection of clinical biomarkers or the clinical use of predictive models, and when annotating behavior or stimuli.

Several practices can help to mitigate the problem of distribution shifts (Dockès *et al*., 2021). One best practice is to start with as large and diverse a training dataset as possible. This diversity should not just reflect the high-level features of primary interest to social neuroscientists, but also the low-level features of the images, text or other data upon which the network is being trained. Care should be taken to avoid selection bias in obtaining the sample: the process for collecting samples should resemble the application context as much as possible.

Once an initial dataset is generated, the robustness of the network can often be improved by data augmentation—programmatic manipulation to expand the training data, increasing its meaningful variability. For example, one could vary the luminance, contrast or other basic features of images, or introduce artificial occlusions. This forces the ANN to learn a more generalizable representation, robust to each of the applied augmentations. Additionally, if a model will be deployed for use on data with different characteristics than the training data, one can use techniques such as importance weighting (Dockès *et al*., 2021) or fine-tuning (Dodge *et al*., 2020) to help mitigate the likely drop in performance.

### Compositionality

Although ANNs perform remarkably well on tasks long associated with human intelligence, such as playing complex games and generating natural language, this does not mean they can do anything a human can do. ANNs tend to perform poorly on tasks involving logical reasoning (Rae *et al*., 2021) and conceptual abstraction (Odouard and Mitchell, 2022). This may be because these tasks require manipulating symbolic systems that are compositional; that is, where the meaning of a statement depends on the meanings of its constituent parts and how those parts are combined (Partee, 1984). When compositional generalization is required, ANNs tend to underperform (Lake and Baroni, 2018). This could explain why the best performing ANN models of human brain activity are trained on tasks that require no symbol manipulation, such as visual object recognition (Lindsay, 2021; Bowers *et al*., 2022; Wichmann and Geirhos, 2023), as opposed to logical reasoning, mathematics or complex communication.

### Explainability

ANNs do not necessarily offer causal or explanatory knowledge. In particular, ANNs are prone to learning misleading ‘shortcuts’: confounds that support prediction, but which are ultimately coincidental or have no causal relationship to the output label (Geirhos *et al*., 2020). Because many phenomena are naturally confounded, it is difficult to guarantee that the predictions of an ANN are based on causally relevant features. In addition to undermining explainability, shortcut learning can lead to generalization failure. For example, ANNs trained to label objects in photographs often produce outputs that depend primarily on subtle variations in background texture rather than on the distinctive features of the objects (Hermann *et al*., 2020). This can result, for example, in images being labeled correctly only when they are photographed in the same environmental context as the training data. Shortcut learning is conceptually related to distribution shift, and can likewise be partially mitigated by using large, diverse datasets.

More generally, it can be difficult to know why an ANN produces any given output. ANNs do not provide tidy lists of beta weights for a set of cleanly separated predictors. Rather, when ANNs make good predictions, we only know that some mixture of features is responsible. Identifying and distinguishing relevant features from confounds requires doing a kind of neuroscience on the network itself, systematically testing how each output depends on a subnetwork of neurons activated by a combination of input features (Olah *et al*., 2018; Samek *et al*., 2019). This process can be laborious and impractical. However, because ANNs are to some extent analogous to biological neural networks, research on how and why ANNs produce their outputs can be a source of neuroscientific knowledge in its own right (Cichy and Kaiser, 2019; Saxe *et al*., 2021). This said, researchers must carefully control experiments to ensure that observed correspondences between ANN models and brain activity are caused by the relevant features of the model and task, and not by incidental choices (Schaeffer *et al*., 2022).

### Social biases

**Fig. 3.**
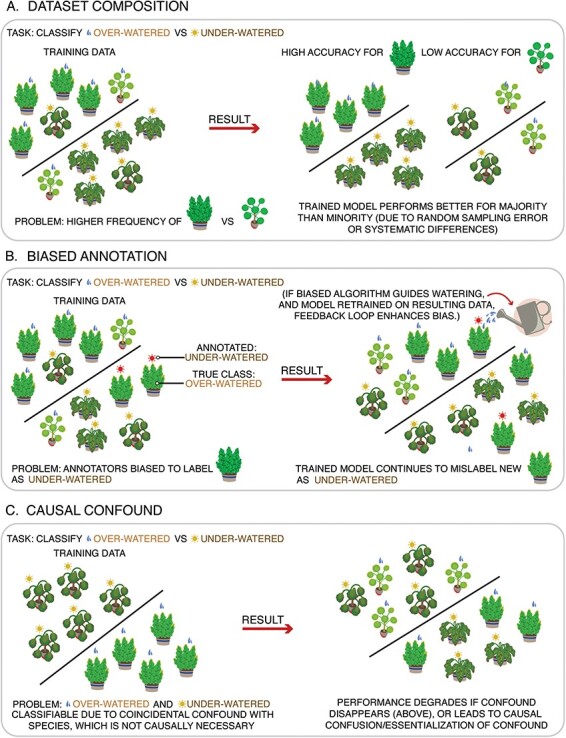
Schematic illustration of different types of bias that ANNs can suffer from, and their origins. (A) When dataset composition is skewed towards one group, that group tends to be annotated more accurately than smaller groups. (B) When humans label ANN training data, any biases in their annotations will influence what the model learns. If the output of a biased ANN is used to guide real world actions, and then those actions can influence the ANN when it is retrained, a feedback loop can enhance this bias. (C) When a confound is present in the data, the ANN may use it as a shortcut to achieve its objective. This can lead to reduced performance when the confound disappears, or cause people to essentialize the confound as a defining feature of the categories being classified. Here, we have illustrated these biases using houseplants to avoid giving the impression that these biases are unique to the social world or to a specific dimension of identity. In the real world, these biases can occur for any such dimension, and often have intersectional features—see the text for examples of how these biases have already affected real world groups.

An important and widely discussed ethical challenge for using ANNs is their propensity to learn and perpetuate human social biases (Birhane, 2021). This can happen several distinct ways, leading to different patterns of bias (Figure 3):

- Dataset composition (Figure 3A): In general, ANNs perform worse on categories that have less training data. This has been a serious problem for models trained on images of human faces (e.g. to perform face recognition), which tend to perform better for majority groups, where training data are easier to acquire, than for minority groups (Buolamwini and Gebru, 2018). This bias is similar to the statistical issue of class imbalance (Johnson and Khoshgoftaar, 2019), except that it applies to imbalances between categories which may not have been measured, but are important for the domain of application. For example, if an algorithm was trained to classify happy versus sad expressions, and there were more happy faces than sad in its training set, this would be an example of class imbalance. Whereas, if there was a balanced number of happy and sad faces, but most of the faces belonged to White people and few to Black people, this would likely yield worse performance for the Black people’s faces, even if race was orthogonal to the emotion labels being classified. This type of bias can be quantified through bias auditing (Mitchell *et al*., 2019). Mitigation practices include building training datasets that are balanced with respect to not just the labels, but also other relevant categories such as demographics, or by importance weighting of undersampled categories (Dockès *et al*., 2021).
- Biased annotation of training data (Figure 3B): Human-created datasets reflect human biases, which are in turn reflected in ANN output. This can happen explicitly, when researchers pay participants to annotate a training dataset, or implicitly, as people discuss the world around them in everyday life. For example, widely held stereotypes manifest themselves in the text that people write on the internet. As a result, when language models are trained on this text, they also express these stereotypes (Stanovsky *et al*., 2019; Charlesworth *et al*., 2021). Much like mitigating human bias, mitigating this form of bias in ANNs is far from a solved problem (Gallegos *et al*., 2023). Approaches range from relatively heavy-handed and fallible ‘manual’ interventions, like using regular expressions to detect and censor problematic outputs, to more integrated solutions, like training secondary bias-detection models and using them to augment the training of large language models.
- Reification: ANNs can be trained using subjective or socially constructed data labels, and the results can be misrepresented or misinterpreted as evidence that those labels are objective facts independent of social context. For example, demographic categories such as race and gender are socially constructed and vary over place and time. Algorithms that label people as belonging to these categories can make these categories seem more immutable and biological than they actually are, effectively ‘laundering’ any bias present in the algorithm (Hutchinson *et al*., 2021; Martinez, 2022). Moreover, when social conditions lead to associations between social groups and socially (un)desirable traits, ANNs can uncritically learn to use these confounds in their predictions, thereby potentially automating the process of generating new stereotypes (Figure 3C). This concern is likely to be particularly relevant to social neuroscientists creating ANN-based biomarkers. Remaining up-to-date on causal inference in machine learning will help to mitigate this bias (Prosperi *et al*., 2020).
- Harmful applications (Figure 3B): The use of biased algorithms can create and reinforce biases in the world. When new data are collected from a population affected by a biased algorithm, this can result in a feedback loop—even a small initial bias can snowball into a highly biased result. For example, consider algorithmic policing (Bennett Moses and Chan, 2018) that directs police to neighborhoods with higher predicted levels of crime, likely reflecting previously existing systemic bias against minority groups. Since police will detect more crime in places where they are deployed than in places where they are absent, they will tend to find more crime in algorithmically predicted neighborhoods, even if crime is uniformly distributed. If this new crime data gets fed back into the algorithmic policing model, a vicious cycle could develop where minority neighborhoods are ever more heavily policed, majority neighborhoods are ignored, and harmful stereotypes are reified. Importantly, even unbiased algorithms can be harmful if applied in biased ways, such as using facial expression analysis algorithms to ‘enhance’ the interrogations of members of a persecuted minority. Social neuroscientists aiming to deploy their ANNs in applied settings should be vigilant about the possibility of feedback loops and unintended or malicious misapplications.

Social neuroscientists must be particularly wary of social biases when using ANNs as annotation tools and when building biomarkers. Biased annotation tools could completely undermine the scientific credibility of a research project. For example, imagine that a researcher used a biased facial expression ANN to annotate action units as people engaged in conversations. Due to some aspect of its training, the algorithm was better at annotating the expressions of White people than those of Black people. The researcher accepts the result uncritically, and concludes that White people have a more expressive conversational style and Black people have a more impassive conversational style. This result would be completely spurious—an artifact of the biased ANN—but it is all too easy to imagine how it might slip into the literature.

Social neuroscientists may be relatively well-equipped to address these issues of social bias, compared with many other fields using ANNs, because the study of bias is already common in the field. An initial step in this process may be to perform a bias audit. By testing a model on a large, unbiased, independent dataset with suitable labels researchers can detect biased model output (Buolamwini and Gebru, 2018; Mitchell *et al*., 2019). After finding biased output, researchers could collect less biased data, retrain their model and try again. However, many social biases are intersectional and context-specific. As such, we discourage readers from thinking that all bias in an ANN has been removed by debiasing it with respect to a single social dimension or a single bias benchmark. Mitigating bias in deep learning will likely remain a major challenge for the foreseeable future.

### Legal issues

Researchers using others’ pretrained ANNs should be aware of relevant intellectual property and privacy concerns. Because it is often difficult to accurately assess the ownership of online content, many datasets used to train ANNs likely contain copyrighted material (Sobel, 2017). This topic merits a more thorough treatment than we can provide here, so we commend you to a devoted guide (Morin *et al*., 2012). Additionally evaluation of risks to privacy may be complicated if trained models are publicly released, as it can be possible to reconstruct training data from model output, potentially leaking sensitive information (Haim *et al*., 2022).

## Conclusion

The rise of deep learning carries great promise for social neuroscience. As we have described here, these algorithms could permit the construction of more useful clinical biomarkers, facilitate more naturalistic experiments and help us build better models of social cognition. However, we have also described the limitations of these algorithms, and some of the thorny practical and ethical issues they create.

We hope we have helped you take a significant step towards understanding the nuts and bolts of ANNs. The goal of this article was to provide you with a high-level conceptual understanding of ANNs that will enable you to engage with them in an informed way as they become more prevalent in the literature. However, this primer was not a technical deep-dive into the math or programming aspects of these models. If this article has motivated you to start using ANNs in your research, you may wish to seek out this level of information as well. Fortunately, there is a rich online ecosystem of tutorials, guides and examples from which you can learn. However, as this ecosystem is constantly evolving, we will point you to a few more academic articles which may prove useful starting points for further study (Goodfellow *et al*., 2016; Kriegeskorte and Golan, 2019; Zhu *et al*., 2019; Mathis *et al*., 2020; Yang and Wang, 2020; Urban and Gates, 2021; Zador *et al*., 2023).

Social neuroscience as a field will need to evolve to meet the needs of researchers who wish to use ANN-based techniques. The level of formal training offered to PhD students in the field is generally insufficient to prepare them to use these methods. Most graduate students take only 1–2 courses in statistics, and the content of these courses has evolved little over the past half-century (Aiken *et al*., 2008). Although students typically gain additional training via an apprenticeship model within their labs, this model cannot easily accommodate the introduction of new techniques with which more senior lab members may be unfamiliar. Until the graduate training catches up to the current needs, programs such as Neuromatch Academy, NeuroHackademy, Methods In Neuroscience at Dartmouth (MIND), the Cajal Advanced Neuroscience Training Programme and the International Max Planck Research Schools (IMPRS) may help fill the gap (Achakulvisut *et al*., 2021; van Viegen *et al*., 2021).

Social neuroscience will also need to carefully consider its collaboration, resource and incentive structures. Large collaborations will be necessary to collect neuroimaging data on the scale necessary to train ANN biomarkers. Resources including data, code and pretrained models must not only be shared, but documented in a way that makes them easy to use. Looking towards examples such as Hugging Face, a popular model repository (Wolf *et al*., 2019), may help guide such developments. Finally, incentive structures need to be reevaluated to place greater value on the activities that support deep learning. The production and curation of large, high-quality datasets and the development and maintenance of useful software tools will be ever more valuable to the field moving forward. These are the essential infrastructure upon which the field advances. Recognizing the value of these activities, particularly in contexts such as hiring and grant funding, will be essential.

Social neuroscience has progressed through several distinct paradigms. At first, the field consisted primarily of systems neuroscientists studying neural behavior in non-human animals. When functional neuroimaging arose, this spurred the emergence of a companion paradigm focused on humans: social cognitive neuroscience (Ochsner and Lieberman, 2001). A decade later, techniques such as multivariate decoding, representational similarity analysis and network models ushered in a new generation of social neuroscience research that moved beyond functional localization and univariate contrasts. Now, ANNs stand poised to catalyze several major movements in the field, from making biomarkers more accurate, to facilitating scalable research on naturalistic social interactions, to serving as a framework for flexible, non-linear cognitive modeling. By supporting and connecting these ongoing advances, ANNs are setting the stage for the emergence of a new paradigm: deep social neuroscience.
